# Supplementary figures and images for: Structural Diversities and Phylogenetic Signals in Plastomes of the Early-Divergent Angiosperms: A Case Study in Saxifragales
Source: Plants (Basel). 2022 Dec 15;11(24):3544. doi: 10.3390/plants11243544 (PMC9787361; doi:10.3390/plants11243544)

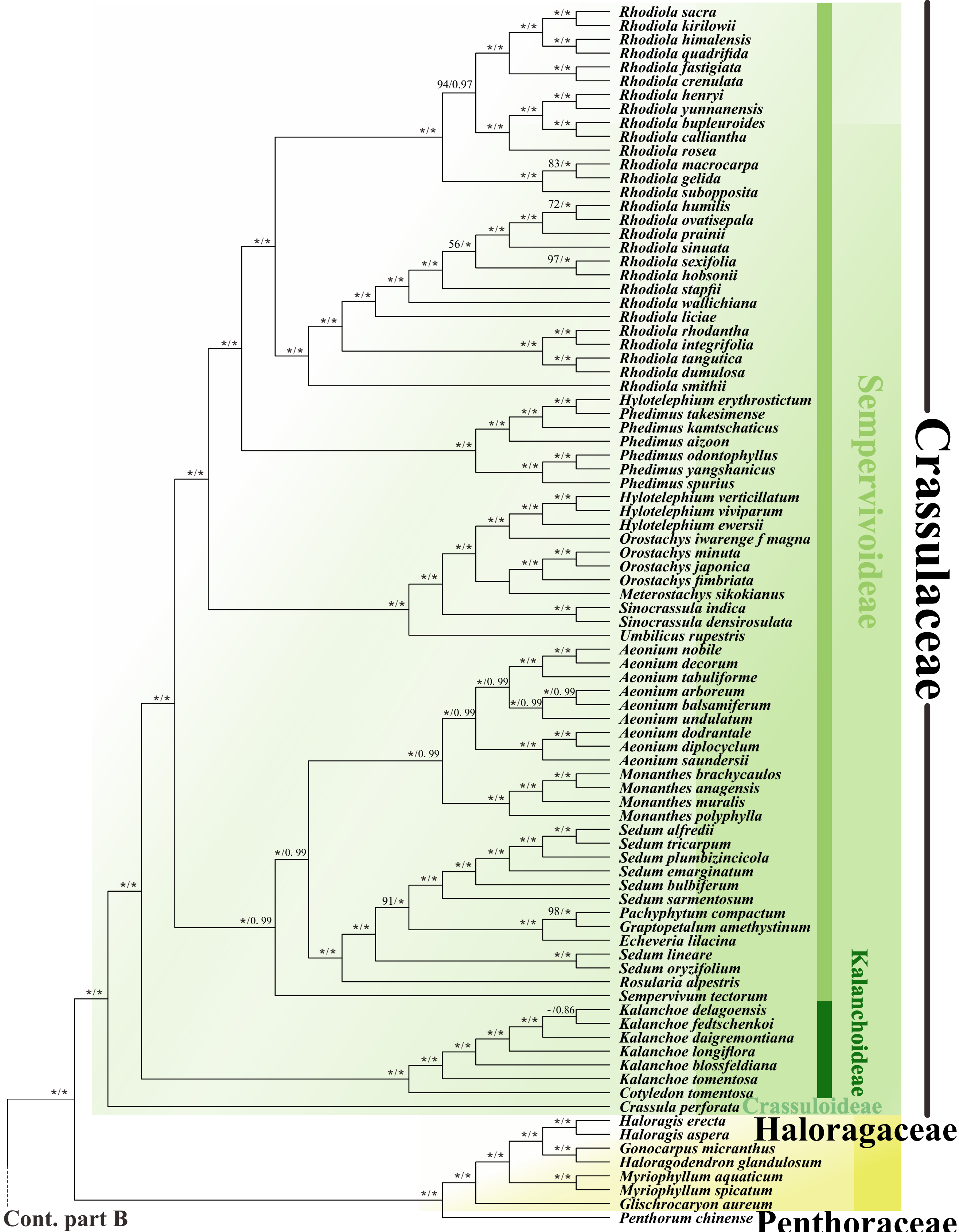

Cont. part A

Cont. part C

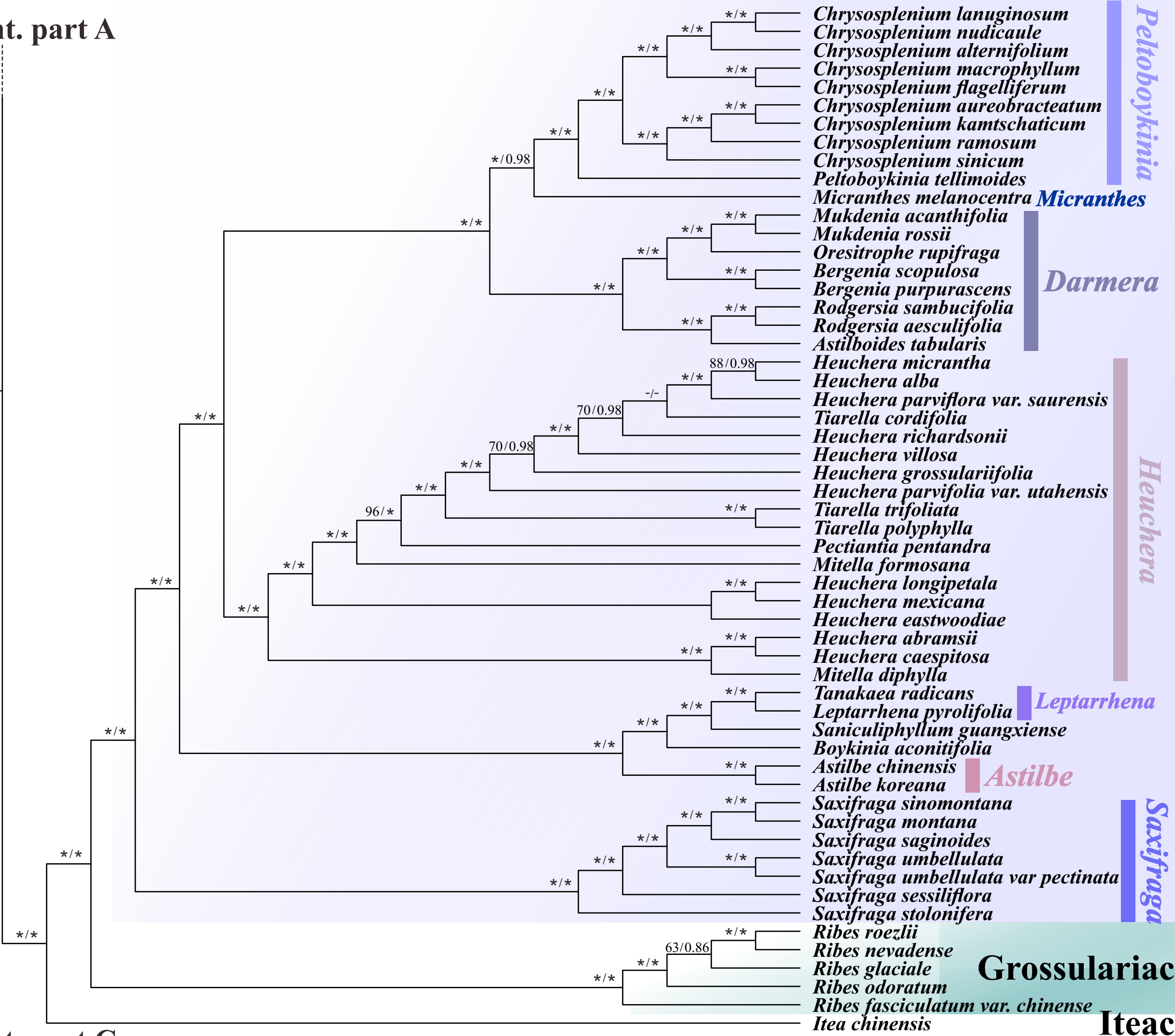

Saxifragaceae

Grossulariaceae

Iteaceae

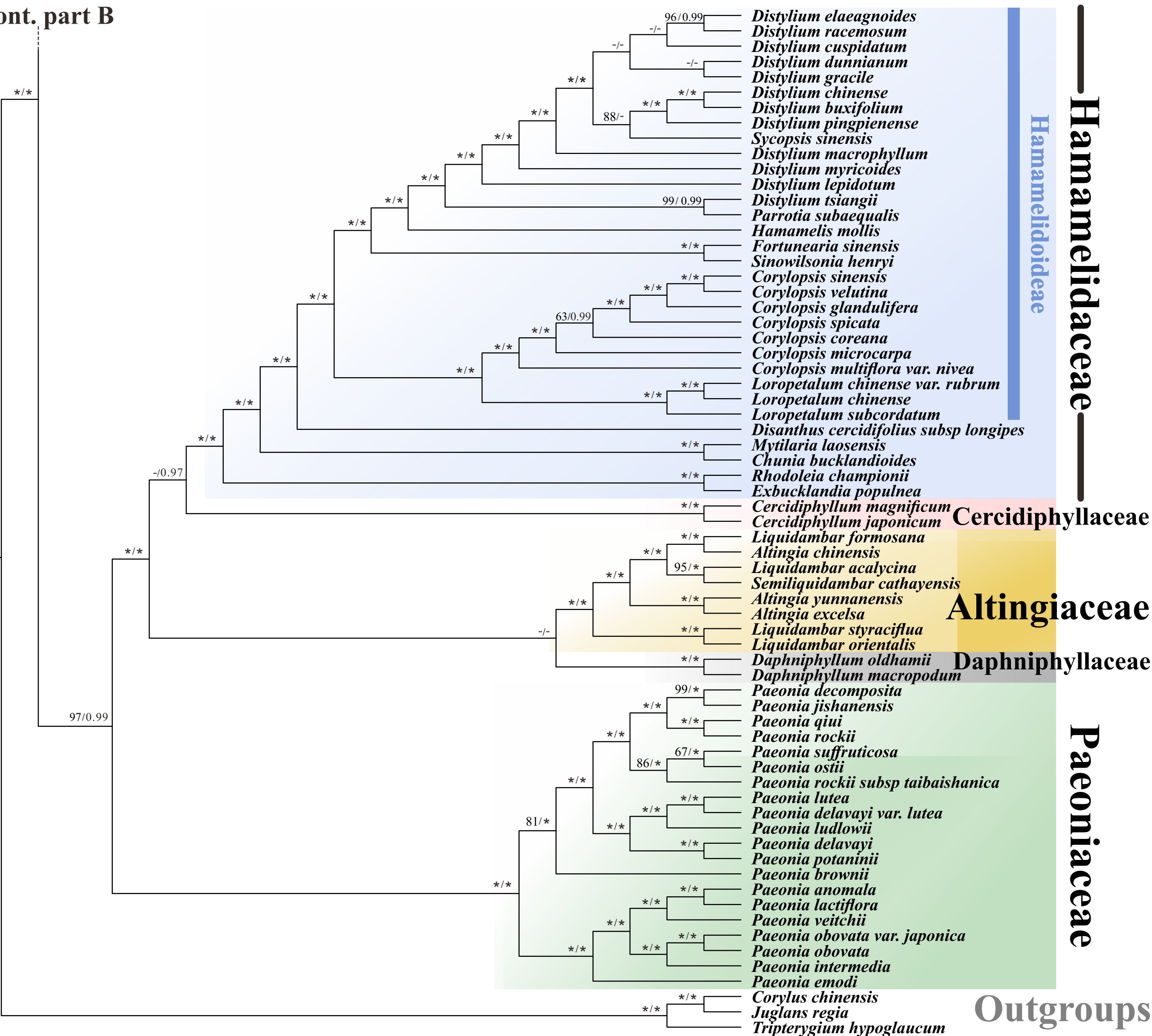

Supplement: Supplementary file 1 [file plants-11-03544-s001.zip › plants-2067334 Figure S1.pdf]
